# The complete chloroplast genome of weedy rye *Secale cereale* subsp. *segetale*

**DOI:** 10.1080/23802359.2022.2080600

**Published:** 2022-06-02

**Authors:** Tianyu Du, Yiyu Hu, Yanqing Sun, Chuyu Ye, Enhui Shen

**Affiliations:** a Shandong (Linyi) Institute of Modern Agriculture of Zhejiang University, Linyi, China; b Institute of Crop Sciences, College of Agriculture and Biotechnology, Zhejiang University, Hangzhou, China; c The Rural Development Academy, Zhejiang University, Hangzhou, China

**Keywords:** Chloroplast genome, weedy rye, *Secale cereale* subsp. *segetale*, phylogenetic analysis

## Abstract

Weedy rye (*Secale cereale* subsp. *segetale* Zhukov 1928) is a problematic weed species in wheat field. However, it can potentially provide valuable genetics resources to increase the genetic variations and introduce desirable genes for rye and wheat breeding. Here, we assembled the complete chloroplast genome of *S. cereale* subsp. *segetale*. The chloroplast genome is 137,051 bp in length, containing a large single copy region (81,090 bp), a small single copy region (12,795 bp) and two separated inverted repeat regions (21,583 bp). A total of 131 unique genes were annotated, consisting of 82 protein-coding genes, 41 tRNA genes, and 8 rRNA genes. The phylogenetic analysis showed that *Secale cereale* subsp. *segetale* (weedy rye) and *S. cereale* subsp*. cereale* (rye) clustered together as sisters to other *Secale* species.

Weedy rye occurs as a weed in cereal fields, mainly in the Near East and Central Asia, and is fully interfertile with cultivated rye (Zohary et al. 2012). Although weedy rye is generally considered to be a malignant weed, as tertiary gene pool of *Triticum aestivum* L. (Santos et al. 2016), it can potentially provide valuable genetic resources (Sun et al. 2022), such as resistance to insects and disease (rust, mildew, aphids, etc.), high yield and resistance to abiotic stress (Che et al. 2008; Hagenblad et al. 2016). In view of climate change and new biotic and abiotic stresses, there is also a need to investigate wild species of rye, which is critical to improve yields and quality of that cereal (Feuillet et al. 2008). Chloroplast genome sequences are useful for understanding plant origin and evolution. However, there is one chloroplast genome in *Secale* currently.

In this study, we assembled the complete chloroplast genome of *S. cereale* subsp. *segetale*. The seed of *S. cereale* subsp. *segetale,* collected in California (38.5475, −121.7393), United States, was acquired from Germplasm Resources Information Network (GRIN) (accession number: CISE 102) and cultured in field (Hangzhou, Zhejiang Provience China). The plant was deposited at Herbarium of Zhejiang University (YuPing Ma, 3160105887@zju.edu.cn) under the voucher number: HZU60244001. Total genomic DNA was sequenced by DNBSEQ-T7 platform. Approximately 80.4 Gbp of clean data was obtained in this study. NGSQCToolkit v2.3 (Patel and Jain 2012) was used for quality control. The clean data was applied in *de novo* assembly by NOVOPlasty v3.6 (Dierckxsens et al. 2017) using the complete chloroplast genome of *Triticum aestivum* (GenBank accession number NC_002762) as a reference. GeSeq online (Tillich et al. 2017) was used for genome annotation. The assembled genome sequences and annotation information have been deposited in GenBank under the accession number LC645358.1.

The total length of *S. cereale* subsp. *segetale* chloroplast genome is 137,051 bp. The genome exhibited a distinct quadripartite structure containing a pair of inverted repeats (IRa and IRb, 21,583 bp each), a large single-copy region (LSC, 81,090 bp) and a small single-copy region (SSC, 12,795 bp). The GC contents of the IR, LSC, and SSC regions are 43.86, 36.22, and 32.17%, respectively. A total of 131 unique genes were annotated, including 82 protein-coding genes, 41 tRNA genes, and 8 rRNA genes.

To understand the phylogenetic relationship between *Secale cereale* subsp. *segetale* and other *Triticeae* species, we built a phylogenetic tree of nine *Triticeae* species based on complete chloroplast genome sequences (NC_024764.1 *Triticum timopheevii*, NC_046698.1 *Triticum zhukovskyi*, NC_024831.1 *Aegilops comosa*, NC_021761.1 *S. cereale*, NC_024831.1 *Aegilops bicornis*, LC_645210.1 *S. strictum* subsp. *kuprijanovii*, LC_649171 *S. sylvestre*, NC_056985 *Hordeum vulgare*) downloaded from NCBI GenBank database. We first performed multiple sequence alignments using MAFFT v7.310 (Katoh et al. 2002) with the parameter ‘–auto –reorder –phylipout’. Then a maximum-likelihood tree was constructed using IQ-tree v1.6.12 (Nguyen et al. 2015) with recommended model TVM + F + I and 1000 bootstrap values. The tree was illustrated and modified by iTOL (Letunic and Bork 2019). The phylogenic tree showed that *S. cereale* subsp. *segetale* was first clustered with *S. cereale* forming as a monophyletic group (Figure 1). *Secale sylvestre* and *S. strictum* subsp. *kuprijanovii* are the wild types of *S. cereal*, and the phylogenetic relationship showed that these wild types may be the progenitor of *S. cereale* subsp. *segetale*. The tree also supported the schematic phylogeny of genus *Secale* rasied by Schreiber et al. (2019). The complete chloroplast genome sequence of *S. cereale* subsp. *segetale* will provide valuable information for genetic studies of *Secale* species.

**Figure 1.**
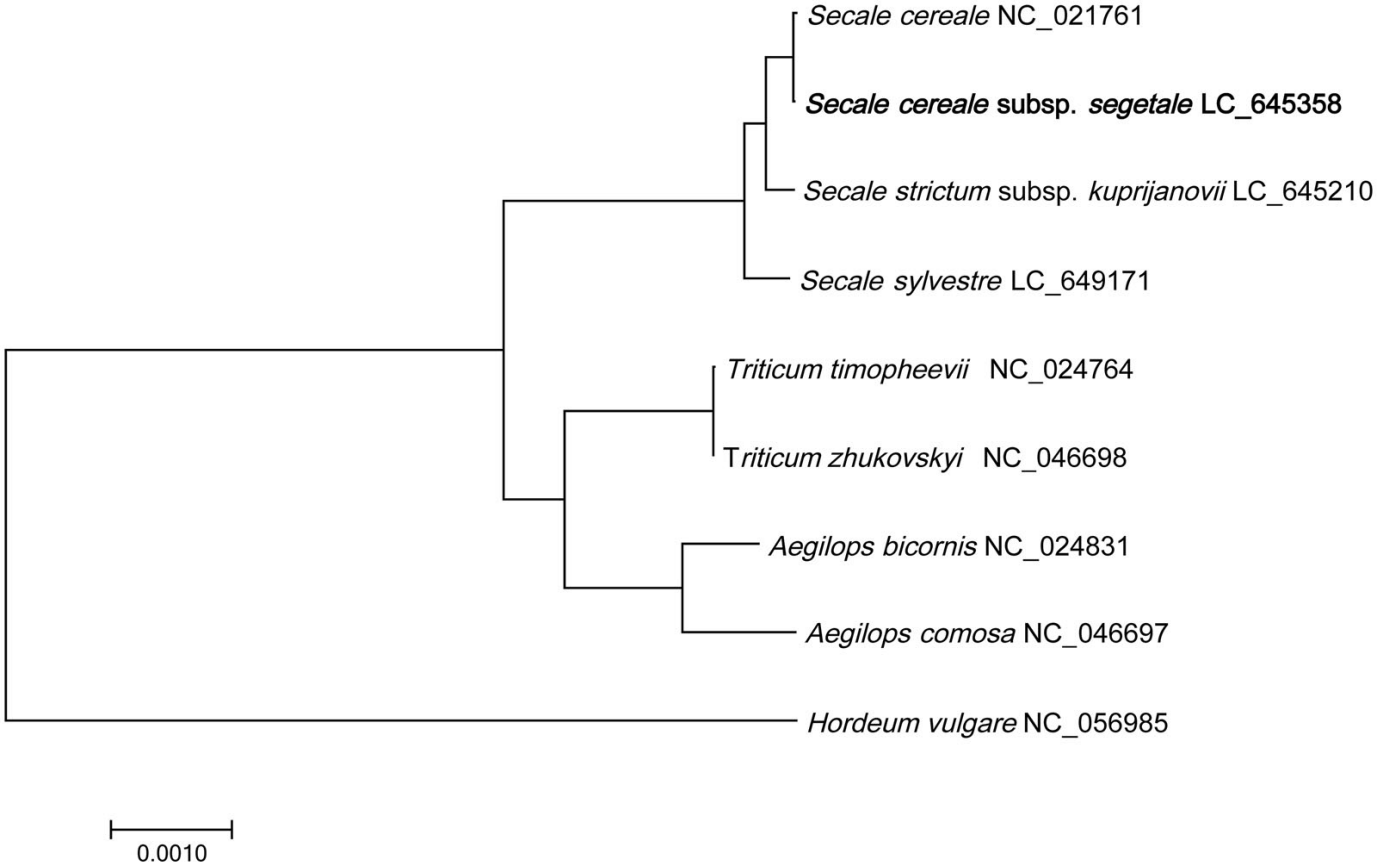
Maximum-likelihood (ML) tree based on 9 *Triticeae* species, using *Hordeum vulgare* as an outgroup. The numbers on the node are the fast bootstrap value based on 1,000 replications.

## Data Availability

The genome sequence data of this study is available in GenBank of NCBI at (https://www.ncbi.nlm.nih.gov) under the accession number LC645358.1. The associated BioProject, SRA, and BioSample numbers are PRJNA749911, SRS9744726 and SAMN20425456 respectively.
